# Retraction: A comprehensive review of deep learning-based single image super-resolution

**DOI:** 10.7717/peerj-cs.621/retraction

**Published:** 2022-10-07

**Authors:** PeerJ Editorial Office

Retraction: Following an investigation by PeerJ Computer Science the authors are in agreement that content in this publication (namely figures and concepts) was inappropriately reused from other published work (Wang, 2020). In consultation with PeerJ Computer Science, the authors request that the article be retracted.
